# C_60_ Fullerene Effects on Diphenyl-N-(trichloroacetyl)-amidophosphate Interaction with DNA In Silico and Its Cytotoxic Activity Against Human Leukemic Cell Line In Vitro

**DOI:** 10.1186/s11671-018-2490-9

**Published:** 2018-03-09

**Authors:** A. Grebinyk, S. Prylutska, I. Grynyuk, B. Kolp, V. Hurmach, T. Sliva, V. Amirkhanov, V. Trush, O. Matyshevska, M. Slobodyanik, Yu. Prylutskyy, M. Frohme, U. Ritter

**Affiliations:** 1 Taras Shevchenko National University of Kyiv, ESC “Institute of Biology and Medicine”, 64 Volodymyrska Str., Kyiv, 01601 Ukraine; 2 Technical University of Applied Sciences Wildau, 1 Hochschulring Str., 15745 Wildau, Germany; 3 Technical University Ilmenau, 25 Weimarer Str., 98693 Ilmenau, Germany; 4 Taras Shevchenko National University of Kyiv, Faculty of Chemistry, Volodymyrska Str., 64, Kyiv, 01601 Ukraine

**Keywords:** Diphenyl-N-(trichloroacetyl)-amidophosphate (HL), C_60_ fullerene, Leukemic CCRF-CEM cells, DNA, Molecular simulation

## Abstract

New representative of carbacylamidophosphates - diphenyl-N-(trichloroacetyl)-amidophosphate (HL), which contains two phenoxy substituents near the phosphoryl group, was synthesized, identified by elemental analysis and IR and NMR spectroscopy, and tested as a cytotoxic agent itself and in combination with C_60_ fullerene.

According to molecular simulation results, C_60_ fullerene and HL could interact with DNA and form a rigid complex stabilized by stacking interactions of HL phenyl groups with C_60_ fullerene and DNA G nucleotide, as well as by interactions of HL CCl_3_ group by ion-π bonds with C_60_ molecule and by electrostatic bonds with DNA G nucleotide.

With the use of MTT test, the cytotoxic activity of HL against human leukemic CCRF-CM cells with IC_50_ value detected at 10 μM concentration at 72 h of cells treatment was shown. Under combined action of 16 μM C_60_ fullerene and HL, the value of IC_50_ was detected at lower 5 μM HL concentration and at earlier 48 h period of incubation, besides the cytotoxic effect of HL was observed at a low 2.5 μM concentration at which HL by itself had no influence on cell viability. Binding of C_60_ fullerene and HL with minor DNA groove with formation of a stable complex is assumed to be one of the possible reasons of their synergistic inhibition of CCRF-CЕM cells proliferation.

Application of C_60_ fullerene in combination with 2.5 μM HL was shown to have no harmful effect on structural stability of blood erythrocytes membrane. Thus, combined action of C_60_ fullerene and HL in a low concentration potentiated HL cytotoxic effect against human leukemic cells and was not followed by hemolytic effect.

## Background

The representative of carbon nanostructure C_60_ fullerene is shown to possess unique physicochemical properties and biological activity not only as antioxidant or photosensitizator but also as modificator of anticancer drugs toxic effect due to its ability to penetrate inside the cell and to function as a drug carrier [1–4]. C_60_ molecule can interact with chemotherapeutic drugs such as doxorubicin, cisplatin, and paclitaxel and can form complexes with them enhancing the therapeutic effect [5–8].

Carbacylamidophosphates (CAPh) are organic molecules which have attracted attention due to its particular structure, biological activity, and perspectives of biomedical application [9–12]. The presence of peptide and phosphoramide groups combined together in the fragment C(O)N(H)P(O) of the molecule determines its interaction with biological molecules and cell membranes. Variation of substituents near phosphoryl and carbonyl groups gives possibility to modulate CAPh stereochemical and pharmacological properties. In particular, different CAPh representatives were shown to possess antineoplastic activity [13, 14].

**Fig. 1:**
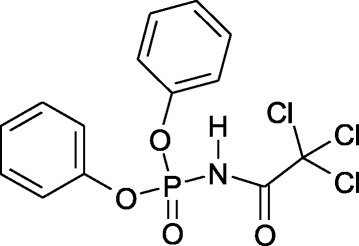
The structure of diphenyl-N-(trichloroacetyl)-amidophosphate (HL)

Recently, we have confirmed that CAPh representative dimethyl-N-(benzoyl)-amidophosphate used in millimolar concentrations range decreased the viability of leukemic L1210 cells and that its toxic effect was facilitated by C_60_ fullerene [15]. We have shown also that introduction of additional aromatic substituents and electronegative CCl_3_ group into CAPh structure resulted in enhancement of its toxicity [16]. Thus, significant toxic effect of dimorfolido-N-trichloroacetylphosphoramide was shown against human leukemic cells of different origin, but its effective concentration was still high and no enhancement of toxicity after combined action with C_60_ fullerene was observed. In continuation of these investigations, we synthesized a new representative of CAPh diphenyl-N-(trichloroacetyl)-amidophosphate (HL) which have two phenoxy substituens instead of morfolido groups near phosphoryl group (Fig. 1).

Тhe aim of the research was to estimate biological activity of diphenyl-N-(trichloroacetyl)-amidophosphate (HL) alone or in combination with C_60_ fullerene with the use of in silico analysis of their interaction with DNA and in vitro study of cytotoxic effects against human leukemic cell line.

## Methods/Experimental

### Chemicals

RPMI 1640 liquid medium, Fetal Bovine Serum (FBS), Penicillin/Streptomycin and L-gluthamine (Biochrom, Germany), Dimethylsulfoxide (DMSO) (Carl Roth GmbH+Co, Germany), MTT [3-(4,5-dimethylthiazol-2-yl)-2,5-diphenyl tetrazolium bromide] (Sigma-Aldrich Co, Ltd., USA), HCl (Kharkivreachim, Ukraine).

### Characterization of Chemical Compound

**Fig. 2:**
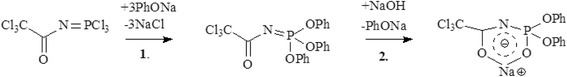
Scheme of solubility of diphenyl-N-(trichloroacetyl)-amidophosphate (HL) in sodium salt

To increase solubility, we have obtained sodium salt of diphenyl-N-(trichloroacetyl)-amidophosphate (HL) according to following reactions (Fig. 2).

The trichlorophosphazothrichloroacetyl solution (0.035 M) in 200 ml of chloroform was slowly added to a well-stirred sodium phenolate suspension (0.106 M, 12.3 g) in 150 ml of chloroform (Fig. 2; stage 1). The mixture temperature was not allowed to rise above 40–50 °C. Stirring was continued for about 1 h, and then the solution was heated up to 70 °C and stirred for 20 min at these conditions. The resulting product triphenoxyphosphazothrichloroacetyl was evaporated. Then, 40 ml of 1 M NaOH was added and refluxed for 90 min (Fig. 2; stage 2). A resulting mixture was evaporated. The solid precipitate of sodium HL was washed three times of diethyl ether and recrystallized from *i*-propanole as a white crystalline powder (80% yield). Colorless crystals of NaL·3H_2_O suitable for X-ray analysis were obtained by *i*-PrOH:H_2_O (9:1 *v*/*v*) solution slow evaporation. The compound is air-stable, highly soluble in water and alcohols. M.p. 215 °С.

HL was identified by elemental analysis and IR and NMR spectroscopy: elemental analyses (C, H, N) were performed using the EL III Universal CHNOS elemental analyzer. IR spectral measurements were performed for samples as KBr pellets on a Perkin–Elmer Spectrum BX FT-IR spectrometer with resolution of 2 cm^− 1^ and accumulations of 8 scans, which are combined to average out random absorption artifactsin the spectral range 4000–400 cm^− 1^. ^1^H NMR spectra in DMSO-d6 solutions were recorded on an AVANCE 400Bruker NMR spectrometer at room temperature.

HL: IR (cm^−1^): 1639 vs, sh (νCO); 1353 s, sh (Amide II); 1194 s, sh (νPO); 941 s, sh (νPN).

^1^H NMR (DMSO-d_6_): 7.05 (t, 2H, γ-CH_2_); 7.205, 7.255 (dt, 8H, α- and β-CH_2_).

For CCl_3_C(O)N(Na)P(O)(OC_6_H_5_)_2_ the elemental composition was determined, %: C 40.58, H 2.35, N 3.15; and calculated, %: C 40.37, H 2.42, N 3.36.

### Synthesis and Characterization of C_60_ Fullerene

A highly stable aqueous colloid solution of C_60_ fullerene (200 μM, purity > 99.5%, nanoparticle average size up to 50 nm) was synthesized in Technical University of Ilmenau (Germany) as described in [17, 18].

### Сell Culture

The experiments were done on human acute T-cell leukemic CCRF-CM cell line. Cell line was purchased from the Leibniz Institute DSMZ-German Collection of Microorganisms and Cell Cultures: CCRF-CM (ACC 240). Cells were cultured in RPMI 1640 medium supplemented with 10% FBS, 1% Penicillin/Streptomycin, and 2 mM Gluthamine, using 25 cm^2^ flasks at a 37 °C with 5% CO_2_ in humidified incubator.

Cells in RPMI 1640 medium were incubated with C_60_ fullerene (16 μM) or HL (2.5, 5, and 10 μM) separately and together during 24, 48, and 72 h. Cell survival without the addition of HL or C_60_ fullerene was received as 100% (control sample contained 0.05 M DMSO).

### Cell Viability (MTT) Assay

Cell viability was assessed by the MTT [3-(4,5- dimethylthiazol-2- yl)-2,5-diphenyl tetrazolium bromide] reduction assay [19]. At indicated time points of incubation, 100 μl aliquots (0,5 × 10^4^ cells) were placed into the 96-well microplates Sarstedt (Nümbrecht, Germany), 10 μl of MTT solution (5 mg/ml in PBS) was added to each well, and the plates were incubated for another 2 h at 37 °C. The culture medium was then replaced with 100 μl of DMSO; diformazan formation was determined by measuring absorption at 570 nm with a microplate reader Tecan Infinite M200 Pro (Männedorf, Switzerland).

### Animals

The study was conducted on white male rats of the “Wistar” line weighing 170 ± 5 g. The animals were kept under standard conditions in the vivarium of the ESC “Institute of Biology and Medicine,” Taras Shevchenko National University of Kyiv. Animals had free access to food and water. All experiments were conducted in accordance with the international principles of the European Convention for protection of vertebrate animals under a control of the Bio-Ethics Committee of the abovementioned institution.

### Estimation of Erythrocytes Hemolysis

Erythrocytes were obtained from heparinized blood of the “Wistar” line rat and diluted in 0.85% NaCl solution to 0.700 o.u. at 630 nm on the Scinco spectrophotometer (Germany). Hemolysis of erythrocytes was caused by 0.001 N HCl. The kinetics of hemolysis was measured spectrophotometrically (*λ* = 630 nm) every 10 s during 2 min. The percentage of hemolysed erythrocytes was calculated as presented in [20]. Erythrocytes were incubated in 0.85% NaCl solution with C_60_ fullerene (16 μM) or HL (2.5 and 10 μM) separately and together during 1 h. Erythrocytes without the addition of HL or C_60_ fullerene were received as 100% (control sample contained 0.05 M DMSO).

### In Silico Study

The double-helix DNA molecule was used as a template from PDB (Protein Data Bank) base. The interaction of DNA molecule with HL separately and in combination with C_60_ fullerene has been studied. We took into consideration the following structures of DNA molecule: 2MIW (CCATCGCTACC - intercalation of compound into a small groove of DNA helix), 1XRW (CCTCGTCC - intercalation of compound into a small groove of DNA helix), and 2M2C (GCGCATGCTACGCG - binding of compound with large and small grooves of DNA helix). We applied the algorithm of systematical docking (SDOCK+), built-in the QXP package (this method demonstrates all possible conformations of the studied structures with the minimal value of Root mean square deviation (RMSD)) [21]. We generated 300 potentially possible complexes with DNA, the ten best of which were selected for the next stage, using a scoring function, built-in the QXP package [22].

The interactions of the DNA molecule with HL separately and in combination with the C_60_ fullerene were characterized by the following parameters: (1) the number of hydrogen bonds, (2) the area of contacting surfaces of DNA and corresponding structure, (3) the distance between the DNA and docked structure, and (4) the total energy of the binding structure.

To assess the stability of the complexes of chemical compound with C_60_ fullerene, we conducted the short molecular dynamics (MD, 100 ps) using Gromacs software tool [23] according to a Nosé-Poincaré-Anderson algorithm (NPA) [24, 25] based on OPLS-AA force field [26, 27].

The calculations were performed on the following parameters: temperature (in Kelvin) - 300; pressure (in kilopascal) - 100; binding involving the hydrogen atom or ligand was limited by the algorithm [25].

### Statistical Analysis

The data were represented as mean ± SD of more than four independent experiments. Mean (M) and standard deviation (SD) were calculated for each group. Statistical analysis was performed using two-way ANOVA followed by post Bonferroni tests. A value of *p* < 0.05 was considered statistically significant. Data processing and plotting were performed by IBM PC using specialized applications GraphPad Prism 7 (GraphPad Software Inc., USA).

## Results and Discussions

### In Silico Study

#### Interaction of HL with DNA

**Fig. 3:**
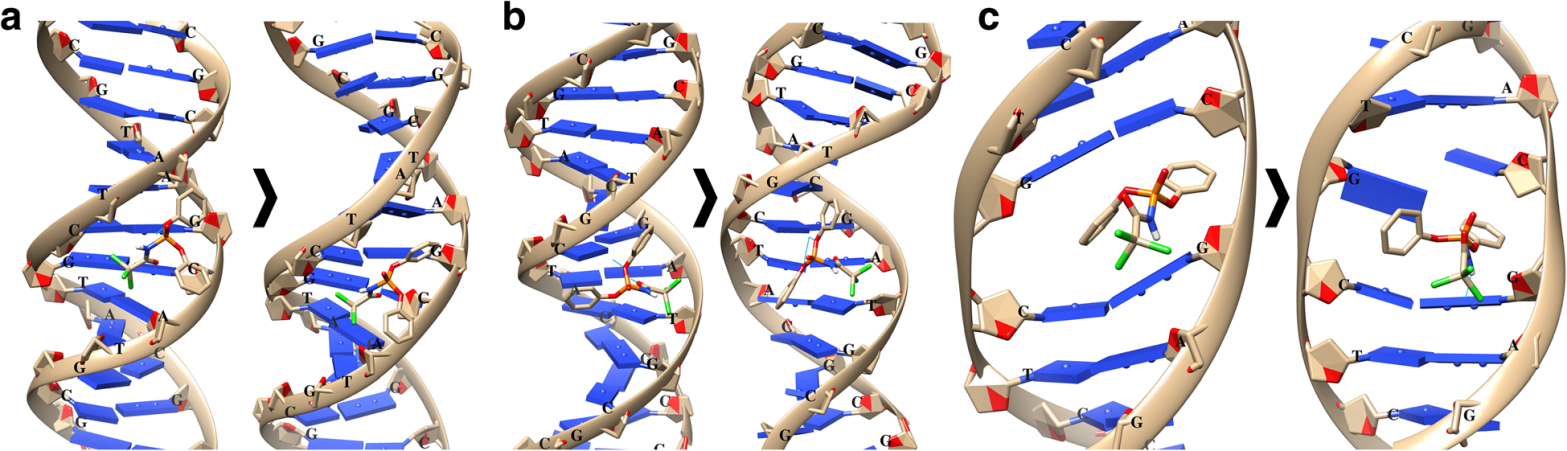
Interaction of diphenyl-N-(trichloroacetyl)-amidophosphate (HL) with DNA molecule: **a**, **b** binding with minor and major grooves; **c** intercalation into a minor groove. The used DNA structure from PDB database: **a**, **b**—2M2C and **c**—1XRW

It was shown that HL could intercalate into DNA and form stable complex when bound in a minor DNA groove via AGC-GCTA nucleotides (Fig. 3a). In this case, the stacking interaction between the nitrogenous base of A nucleotide and HL phenyl group as well as electrostatic interaction between CCl_3_ group and C nucleotide was formed. After MD simulation, the RMSD values for DNA double helix and HL were found to be 3.3 and 1.62 Å, respectively. Nucleotide environment of HL (GCA-CTA) was partly changed and stacking interaction was lost, while a hydrogen bond was formed between G nucleotide and CO group of HL.

Binding of HL with a major DNA groove occurred via TCG-AT nucleotides (Fig. 3b). In this case, a hydrogen bond between the CO group of HL and nitrogenous base of A nucleotide was formed and stacking interaction between both HL phenyls and nitrogenous bases of C and G nucleotides occurred. Besides, a probability of electrostatic bond between HL CCl_3_ group and nitrogenous bases of AT nucleotides appeared.

After MD simulation, no changes in the nucleotide environment of HL were detected. The RMSD values for DNA and HL were 2.77 and 1.58 Å, respectively. Due to it the stacking interaction between C nucleotide and phenyl group disappeared.

In the case of HL intercalation into DNA, its environment consisted of CG-CG nucleotides (Fig. 3c). The stacking interaction with CG nucleotides appeared: one phenyl group was clamped between the nitrogenous bases and the other formed a stacking interaction with C nucleotide.

After MD simulation, the RMSD values for DNA double-helix and HL were 1.71 and 1.89 Å, respectively. The nucleotide environment of HL was not changed. One of the phenyl group formed a ion-π interaction with the nitrogenous base of G nucleotide, which appeared to be shifted by 1.12 Å.

The obtained energy parameters testified that the energy of steric clashes between DNA and HL as well as within HL itself was insignificant (Table 1).

**Table 1:** Energy parameters (kJ/mol) of diphenyl-N-(trichloroacetyl)-amidophosphate (HL) interaction alone and in combination with C_60_ fullerene with DNA double-helix

Structure	The energy parameters				
	FreE	Cntc	Hbnd	Bump	Int
Binding with a major DNA groove					
HL	− 6.6	− 45.1	− 2.0	2.5	6.3
HL+С_60_	− 23.1	− 75.7	− 2.3	8.8	5.0
Binding with a minor DNA groove					
HL	− 13.1	− 56.8	− 1.8	2.5	3.6
HL+С_60_	− 23.3	− 84.7	0.0	7.8	6.8
Intercalation into a minor DNA groove					
HL	− 8.0	− 62.2	− 1.0	6.2	8.7
HL+С_60_	− 8.0	− 62.2	− 1.0	6.2	8.7
С_60_+HL	− 23.5	− 100	0.0	20.0	5.2

*FreE* the total energy of compounds binding with DNA; *Cntc* the contact energy of compounds interaction with DNA; *Hbnd* the energy of hydrogen bonds; *Bump* the energy of steric clashes between DNA and build-in compounds; *Int* the energy of steric clashes between the atoms of build-in compounds

We have done the comparative analysis of energy parameters of HL binding with minor and major DNA grooves or its intercalation into minor DNA groove. Bump values were shown to be 6.2 kJ/mol in the case of HL intercalation into DNA and 2.5 kJ/mol in the case of its binding with minor and major DNA grooves (Table 1). Int values were 8.7 kJ/mol in the case of HL intercalation into DNA, 6.3 kJ/mol in the case of its binding with a major DNA groove, and 3.6 kJ/mol in the case of its binding with a minor DNA groove. These data showed that HL binding with a minor groove of DNA was the most stable.

#### Combined Interaction of HL and C_60_ Fullerene with DNA

**Fig. 4:**
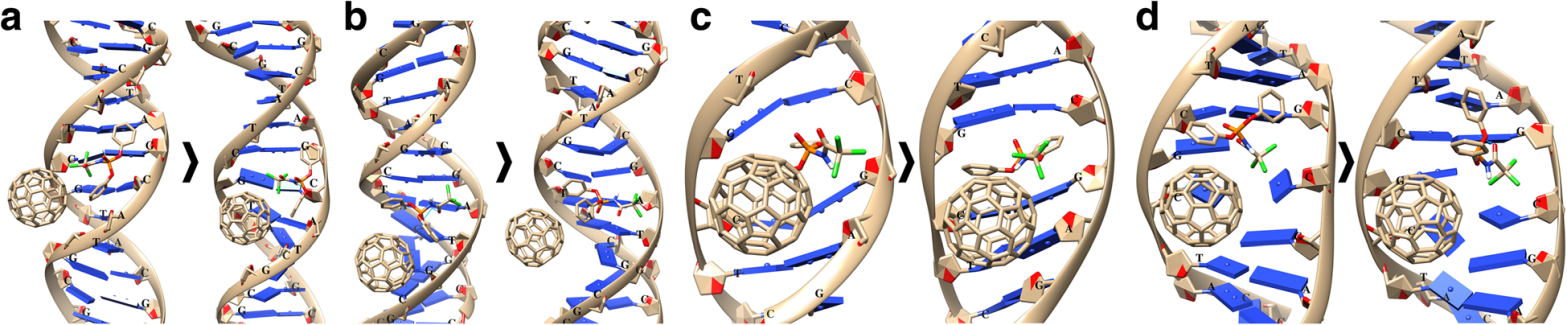
Combined interaction of diphenyl-N-(trichloroacetyl)-amidophosphate (HL) and C_60_ fullerene with DNA (HL+C_60_ or C_60_+HL versions): **a**, **b** binding with minor and major grooves, **c**, **d** intercalation into minor and major grooves. The used DNA structures of the PDB database: **a**, **b**—2M2C, **c**—1XRW, and **d**—2MIW

Previously with the use of computer simulation, we have demonstrated that C_60_ molecule could interact with DNA and form a stable C_60_+DNA complex when binding with DNA minor groove [15]. As it is shown in Fig. 4, C_60_ fullerene could also form ion-π bonds with CCl_3_ groups of HL molecule.

We have used two versions of molecular modeling of HL, C_60_ fullerene and DNA interaction, which were proposed in [16] and proved to be useful for interpretation of MD simulation results. We have used 1XRW PDB structure of DNA molecule in the version, when initially HL and then C_60_ molecule intercalate into DNA (HL+C_60_) and 2MIW PDB structure of DNA molecule - in the version, when initially C_60_ molecule and then HL intercalate into DNA(C_60_+HL).

The binding with a minor DNA groove in the case of HL+C_60_ version occurred via GCTA-GCAT nucleotides (Fig. 4a). Phenyl groups filled a minor groove and entered into stacking interactions: one group with C_60_ fullerene and the other one with the nitrogenous base of G nucleotide. The electrostatic interactions arose between CCl_3_ group of HL and both the nitrogenous base of G nucleotide and C_60_ fullerene.

According to MD simulation results, a DNA double helix in this case was characterized by considerable mobility (RMSD value is 3.08 Å), RMSD value for HL was 2.04 Å, while C_60_ fullerene remained virtually immovable. As a result, the nucleotide environment of HL+C_60_ structure was changed by TGC-GCATG. Besides, a hydrogen bond between amino group of HL and DNA and stacking interactions between the nucleotides of nitrogenous GC bases and C_60_ fullerene appeared.

The binding of HL+C_60_ with a major DNA groove occurred via C-ATCC nucleotides (Fig. 4b). Hydrogen bonds were formed between HL CO group and the nitrogenous base of A nucleotide. Phenyl groups filled a major DNA groove and entered into stacking interaction with C_60_ fullerene.

According to MD simulation results, the nucleotide environment of HL+C_60_ structure was not changed: the values of RMSD for DNA and HL were 3.14 and 2.24 Å, respectively, C_60_ fullerene remained virtually immovable. In this case, all interactions between HL and C_60_ fullerene disappeared and HL sterically interacted only with DNA. It is supposed that as a result C_60_ fullerene as well as HL would be pushed out from the major DNA groove.

When HL+C_60_ were intercalated into a minor DNA groove (Fig. 4c), the binding with CGT-GAG nucleotides occurred. C_60_ fullerene occurred to be built into a minor DNA groove and to interact with it sterically. HL phenyl groups formed stacking interactions with the nitrogenous bases of CG-CG nucleotides. According to MD simulation, the values of RMSD for DNA and HL were 2.29 and 2.13 Å, respectively, C_60_ fullerene remained virtually immovable, and CC1_3_ group of HL entered into electrostatic interaction with the nitrogenous base of G nucleotide.

In the case of C_60_+HL version, the binding with a minor DNA groove (Fig. 4d) occurred via CGC-GCC nucleotides. One of the HL phenyl groups formed a stacking with C_60_ fullerene and the other with G nucleotide. The CC1_3_ group of HL appeared to form electrostatic bond with the nitrogenous base of C nucleotide. According to MD analysis, the values of RMSD for DNA and HL in this case were 2.35 and 2.75 Å, respectively, C_60_ fullerene stayed immovable, and the nucleotide environment of C_60_+HL structure was changed by CGCT-GC. In addition, the C_60_ molecule penetrated deeper into DNA and formed a stacking interaction with the nitrogen base of C nucleotide.

According to the calculated energy parameters, the complex formed in the case of C_60_+HL intercalation into a minor DNA groove was the most rigid (the Bump value 20.0 kJ/mol) (Table 1). In contrast in the case of HL+C_60_ interaction with DNA, this parameter was only 6.2 kJ/mol when HL+C_60_ was intercalated into a minor DNA groove, 7.8 kJ/mol when it was bound with a minor DNA groove, and 8.8 kJ/mol when it was bound with a major DNA groove. Besides, the energy parameters showed that formation of strong hydrogen bonds inside HL+C_60_+DNA complex was possible only in the case of binding with a major groove of DNA, when the value of Hbnd was − 2.3 kJ/mol, in other types of interaction it was equal zero or − 1.0 kJ/mol (Table 1). Moreover, according to MD results in this case both C_60_ fullerene and HL appeared to be displaced from a major DNA groove.

Therefore, C_60_+HL binding with a minor DNA groove is suggested to be the most probable version of C_60_ fullerene, HL, and DNA combined interaction.

### In vitro Study of HL Biological Effects

#### CCRF-CЕM Cells Viability

In in vitro experiments, the long-term influence of diphenyl-N(trichloroacetyl)-amidophosphate (HL) in the range 2.5–10 μM on the viability of human leukemic CCRF-CЕM cells was estimated by MTT test. Cells were incubated for 24, 48, and 72 h in RPMI 1640 medium with either 16 μM C_60_ fullerene or HL alone or their combination. Viability of cells incubated without C_60_ or HL was considered to be 100%.

**Fig. 5:**
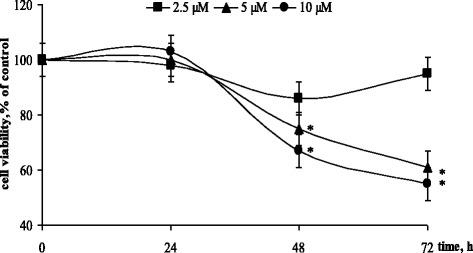
Viability of CCRF-CEM cells incubated with diphenyl-N-(trichloroacetyl)-amidophosphate (HL) in different concentrations. (M ± m, *n* = 8); *p < 0.05 compared to control cells

At 24 h of incubation, no effect of HL on CCRF-CЕM cells viability was observed (Fig. 5). Still at more prolonged incubation, the cytotoxic activity of HL in 5 and 10 μM concentration became obvious, cell viability at 48 h was inhibited by 25 and 33%, respectively, and continued to fall at 72 h.

It should be noted that 50% decrease of CCRF-CЕM cells viability (IC_50_) was detected at 72 h under the action of HL in 10 μM concentration (Fig. 5). Recently, we have shown that another CAPh representative dimorfolido-N-trichloracetylphosphorylamid caused 50% decrease of CCRF-CЕM cells viability at 72 h in concentration 1 mM [16]. The comparative analysis of these data demonstrates that introduction of phenoxy instead of morfolido groups into the structure of CAPh derivative allowed to decrease by two orders its effective toxic concentration against leukemic cells. We assume that conformational flexibility of –P(O)(OC_6_H_5_) core ensured more effective interaction of this compound with DNA.

**Fig. 6:**
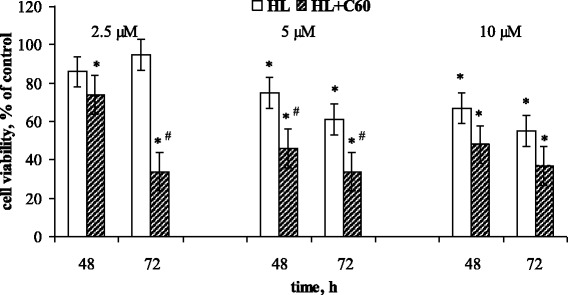
Viability of CCRF-CEM cells incubated with diphenyl-N-(trichloroacetyl)-amidophosphate (HL) alone or in combination with 16 μM C_60_ fullerene. (M ± m, *n* = 8); *p < 0.05 compared to control cells; ^#^*p* < 0.05 compared to HL

No influence of C_60_ fullerene used alone on cell viability during incubation period was detected (data not presented). At the same time, the results shown on Fig. 6 demonstrate that C_60_ fullerene intensified HL cytotoxic activity against CCRF-CЕM cells. Under combined action of C_60_ fullerene and HL, 50% decrease of cell viability was observed at lower HL concentration (5 μM) and at earlier period (48 h) of incubation than under the action of HL by itself. Moreover at 72 h of combined action of C_60_ fullerene and HL, the cytotoxic effect of HL was detected in a low 2.5 μM concentration at which HL by itself had no influence on cell viability (Fig. 6).

Thus, C_60_ fullerene was shown to potentiate cytotoxic effects of HL and to increase significantly leukemic cells sensitivity to its action in a low concentration. Taking into account that C_60_ fullerene is able to accumulate inside leukemic cells over 24 h [28] and to localize in intracellular compartments [29–31], particularly in the nucleus [32, 33], its interaction with nuclear DNA of actively proliferated cancer cells should not be excluded.

So the data obtained in vitro are in accordance with the results of in silico study and demonstrate that initial binding of C_60_ molecule and then of HL with minor DNA groove with formation of a stable complex could be one of the possible reasons of their synergistic inhibition of CCRF-CЕM cells proliferation.

#### Erythrocytes Resistance to Hemolysis

On estimation of anticancer potential of HL and C_60_ fullerene combination, it is important to take into account its possible effects on nonmalignant cells, in particular on blood cells.

**Fig. 7:**
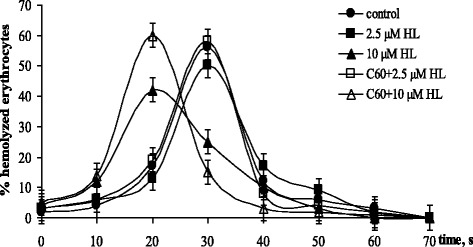
Erythrocytes resistance to hemolysis in the presence of diphenyl-N-(trichloroacetyl)-amidophosphate (HL) separately and in combination with 16 μM C_60_ fullerene. (M ± m, *n* = 8)

Study of erythrocytes resistance to acidic hemolysis allows to elucidate the influence of pharmacological agent at the membrane level. Dynamics of hemolysis reflects the dynamics of erythrocyte plasma membrane disruption and hence the stability of its structural organization. In Fig. 7, the dependence of the percentage of hemolysis erythrocytes were incubated for 1 h in NaCl solution without additions (control) and with either HL or C_60_ fullerene alone or in combination. No influence of 16 μM C_60_ fullerene on erythrocytes hemolysis was detected (not shown). HL in 2.5 μM concentration alone or in combination with C_60_ affected erythrocytes resistance to hemolysis (Fig. 7). Meanwhile, under the action of HL in 10 μM concentration acceleration of hemolysis with maximum at 20 s was detected. Combined action of 10 μM HL and C_60_ fullerene was followed by further hemolysis intensification with 60% of hemolysed erythrocytes at 20 s.

The data obtained showed that application of C_60_ fullerene in combination with 2.5 μM HL had no harmful effect on structural stability of blood erythrocytes membrane and at the same time allow to increase significantly cytotoxic activity of HL in this low concentration against leukemic cells. Despite the synergetic cytotoxic effect of C_60_ fullerene and HL in 10 μM concentration against leukemic cells, the application of their combination appeared to be limited by intensification of hemolytic effect.

Finally, in the context of the above in vitro study, it is important to emphasize that a water/solid interface have confined water which also can affect both the transport, thermodynamic properties of nanostructures [34, 35], and their interaction with cell membranes [36].

There are literature data on the intracellular localization of carbacylamidophosphates derivatives, in particular, penetration of phosphoramidates into the cells. Thus, phosphoramidate derivatives can penetrate throw the membrane of MDA-231 breast cancer and lung cancer (H460, H383, and H2009) cell lines [37]. Nitrobenzyl phosphoramide mustards were shown to permeate across cell membranes and to be localized in mitochondria of NTR^+^ mammalian cells [10]. It is not excluded that C_60_ fullerene, which is able to penetrate the membrane of the cancer cell due to passive diffusion or endocytosis [28, 30, 32] with accumulation in the nucleus and mitochondria [30, 32, 33], could be a transporter of small antitumor molecules [38–40].

## Conclusions

A new representative of carbacylamidophosphates derivatives which have two phenoxy substituens near phosphoryl group diphenyl-N-(trichloroacetyl)-amidophosphate (HL) was synthesized and tested as a cytotoxic agent itself and in combination with C_60_ fullerene. According to molecular simulation results, when C_60_ fullerene and then HL were bound with a minor DNA groove, a rigid complex was formed stabilized by stacking interactions of HL phenyl groups with C_60_ fullerene and DNA G nucleotide, as well as by interactions of HL CCl_3_ group by ion-π bonds with C_60_ molecule and by electrostatic bonds with DNA G nucleotide.

With the use of MTT test, we have shown cytotoxic activity of HL against human leukemic CCRF-CM cells with IC_50_ value detected at 10 μM concentration at 72 h of cells treatment. The cytotoxic effect of HL was facilitated by C_60_ fullerene. Under combined action of 16 μM C_60_ fullerene and HL, the value of IC_50_ was detected at lower 5 μM HL concentration and at earlier 48 h period of incubation and besides the cytotoxic effect of HL was observed at a low 2.5 μM concentration at which HL by itself had no influence on cell viability.

Previously, we have shown a significant toxic effect of dimorfolido-N-trichloroacetylphosphoramide against human leukemic cells of different origin, but its effective concentration was high and no enhancement of toxicity after combined action with C_60_ fullerene was observed [16]. We assume that introduction of flexible of –P(O)(OC_6_H_5_) groups instead of morfolido groups into CAPh derivative contributed to lowering of its toxic concentration against human leukemic cells ensuring its effective interaction with C_60_ molecule and DNA. Binding of C_60_ fullerene and HL with minor DNA groove with formation of a stable complex is assumed to be one of the possible reasons of their synergistic inhibition of CCRF-CЕM cells proliferation.

We have revealed that application of C_60_ fullerene in combination with 2.5 μM HL had no harmful effect on structural stability of blood erythrocytes membrane, while combination of C_60_ fullerene with 10 μM HL appeared to be limited by intensification of hemolytic effect.

Thus, C_60_ fullerene was shown to potentiate cytotoxic effect of HL and to increase significantly human leukemic cells sensitivity to its action in a low concentration. A combination of C_60_ fullerene with HL in a low concentration 2.5 μM may be promising for further biomedical studies.

## Abbreviations

DMSO: Dimethyl sulfoxide
DNA: Deoxyribonucleic acid
FBS: Fetal bovine serum
HL: Diphenyl-N-(trichloroacetyl)-amidophosphate
IR: Infrared spectroscopy
MD: Molecular dynamics
MTT: 3-(4,5-Dimethyl-2-thiazolyl)-2,5-diphenyl-2-H-tetrazolium bromide
NMR: Nuclear magnetic resonance
RPMI - 1640 medium: Roswell Park Memorial Institute medium
